# Cheloid

**Published:** 1880-05-20

**Authors:** Lindsay Jonhson

**Affiliations:** Demonstrator of Anatomy in Southern Medical College of Atlanta, Ga.


### *KELOID OR CHELOID.*

BY LINDSAY JONHSON, M.D., DEMONSTRATOR OF ANATOMY IN SOUTHERN MEDICAL COLLEGE OF ATLANTA, GA.

Having met in practice a case of rare affection, first described by Alibert under the name of cancroid, and subsequentiy under that of Cheloid or Keloid, from the fancied resemblance of the disease to a tortoise, I thought that a brief history of this particular case would be acceptable to the many readers of your popular journal.

That the patient in question possessed a peculiar *diathesis* which developed the disease, I have not the slightest doubt. This fact I shall endeavor to make incontrovertible; and this fact, perhaps more than anything else, influenced me to attempt a history of the case.

During the harvesting season of 1878, the patient often having bound and handled wheat for two days, became rather suddenly and excessively annoyed by an intolerable itching and burning of skin over the chest, the intensity of which lasted for hours, notwithstanding every and all available means were employed for its relief. The severity of the symptoms having passed off, nothing more was done, all present agreeing that the catastrophe was due to the irritation caused by the rubbing of the wheat straw against his breast, while engaged in forming the wheat into bundles; nor was the laceration of the skin by the finger nails in the act of scratching considered worthy of notice.

Not more than a fortnight after this the patient came to my office, and after exhibiting to view the cause of his anxiety, gave the above history.

Immediately over the middle sternal bone was situated an irregular mass of outgrowth, smooth, shining, glistening, and presenting very much the appearance of the scar of a burn. From the center of this mass, certainly an outgrowth of the fibro-cellular tissue of the skin, sprang processes or prolongations of contracted tissue, radiating in various directions, affording the whole a configuration of the devil-fish.

I have said that the disease in this case was the result of a peculiar diathesis, and base my position upon the subsequent history of the case. Prior to the appearance of this keloid affection, the patient had been entirely free from anything that could suggest the possibility of such a condition. His morality was proverbial, and the possibility of venerial positively excluded—save hereditary transmission—a thing hardly to be considered.

Well, now in the first place, I took a clean, new bistoury, made several scratches on his arm, about three inches above the elbow, exciting slight irritation, in some places going deep enough to draw blood. I then gave a placebo and instructed him to return in three weeks. He did so, and, to my utter astonishment, the very same keloid condition existed here as upon his breast, though hardly to such a degree. Not yet satisfied, I prevailed upon him to let me repeat the operation elsewhere, promising him treatment gratis. Suffice it to say the same condition had supervened as existed upon the breast and arm.

I will not go further into the history of the disease, nor of this case, than to say that the result of treatment in this instance refutes the general belief that the disease is incurable.

The treatment pursued was strictly alterative and tonic, with the local application of emollient and absorbent unguents. Internally I administered iodoform and quinia; iodoform and ferrum reduc., alternating with hydg. biniodid and extract sarsaparilla fl., at the same time using locally ung. iodinii co. et ung. petrolei, partes equales, well rubbed on twice daily.

The result was altogether satisfactory, not the slightest manifestation of the disease existing at the end of four months, and up to the present there is still no indication that the affection will ever return.

In this article I have refrained from anything like a history of the disease as described by Alibert, nor have I thought it necessary to enter into full details as to the causes—traumatic and idiopathic—which give rise to the disease, except in the subject-case.

That the affection was a veritable keloid I think no one will doubt, and that keloid is not incurable under the given line of treatment, I further think is established beyond any peradventure.

